# The Mechanism of Ferroptosis Regulating Granulosa Cell Apoptosis and Oxidative Stress Through the NF-κB/PTGS2 Axis in Porcine Atretic Follicles

**DOI:** 10.3390/antiox14091071

**Published:** 2025-09-01

**Authors:** Yiting Yang, Yuxu He, Mailin Gan, Xue Zhao, Tianci Liao, Yuhang Lei, Lei Chen, Lili Niu, Ye Zhao, Yan Wang, Linyuan Shen, Yihui Liu, Li Zhu

**Affiliations:** 1 State Key Laboratory of Swine and Poultry Breeding Industry, College of Animal Science and Technology, Sichuan Agricultural University, Chengdu 611130, China; yangyiting0914@stu.sicau.edu.cn (Y.Y.); h2021202015@stu.sicau.edu.cn (Y.H.); ganmailin@sicau.edu.cn (M.G.); zhaoxue@stu.sicau.edu.cn (X.Z.); 2020202019@stu.sicau.edu.cn (T.L.); leiyuhang@stu.sicau.edu.cn (Y.L.); chenlei815918@sicau.edu.cn (L.C.); niulili@sicau.edu.cn (L.N.); zhye@sicau.edu.cn (Y.Z.); wangyan2023@sicau.edu.cn (Y.W.); shenlinyuan@sicau.edu.cn (L.S.); 2 Key Laboratory of Livestock and Poultry Multi-Omics, Ministry of Agriculture and Rural Affairs, College of Animal Science and Technology, Sichuan Agricultural University, Chengdu 611130, China; 3 Farm Animal Genetic Resources Exploration and Innovation Key Laboratory of Sichuan Province, Sichuan Agricultural University, Chengdu 611130, China; 4 Key Laboratory of Bio-Resource and Eco-Environment of Ministry of Education, Animal Disease Prevention and Food Safety Key Laboratory of Sichuan Province, College of Life Sciences, Sichuan University, Chengdu 610065, China; 5 Sichuan Province General Station of Animal Husbandry, Chengdu 610066, China

**Keywords:** ferroptosis, melatonin, NF-κB, PTGS2, oxidative stress

## Abstract

Ferroptosis is a new mode of cell death, which is characterized by inducing the accumulation of lipid peroxides dependent on iron ions and reactive oxygen species. It has been found that ferroptosis can lead to follicle atresia by promoting granulosa cell death and increasing its reactive oxygen species content, but the specific mechanism has not been elucidated. Through transcriptome sequencing, we found that ferroptosis markers and related genes were upregulated in porcine atretic follicles. PTGS2 was found to be differentially expressed between atretic and healthy follicles. By inhibiting NF-κB nuclear translocation, inhibition of the PTGS2 gene expression reduced the degree of ferroptosis in granulosa cells and rescued granulosa cell death and oxidative stress caused by ferroptosis. Therefore, we propose that the NF-κB/PTGS2 axis plays a key role in ferroptosis-induced granulosa cell death, leading to follicular atresia. Melatonin, a neurohormone secreted by the pineal gland of the upper thalamus, is involved in the regulation of various metabolic, immune, reproductive, and other processes. In the ferroptosis treatment group, melatonin treatment alleviated the degree of ferroptosis (downregulation of ferroptosis marker genes and markers) and decreased the expression of PTGS2. In summary, we have demonstrated that melatonin inhibits ferroptosis via the NF-κB/PTGS2 axis in granulosa cells.

## 1. Introduction

The follicle is the basic functional unit of the female mammalian ovary, which has biological functions such as producing mature oocytes, promoting animal reproduction, and synthesizing estrogen [1]. Follicle development includes follicle selection, recruitment, dominant follicle selection, and ovulation. Follicles in different stages are divided into primordial follicles, primary follicles, secondary follicles, and mature follicles [2]. Granulocytes are the largest cell population in the mature follicle, starting from the primordial follicle, accompanying the primordial follicle, and participating in the whole process of follicular development, maturation, and ovulation [3]. According to the location of existence and their own function, they are divided into cumulus granule cells and parietal granule cells. Cumulus granulosa cells mainly play a role in supporting oocyte development and regulating follicle maturation [4]. Pietal granulosa cells can secrete hormone receptors, including follicle-stimulating estrogen (FSH) receptor and luteinizing hormone (LH) receptor, and they are transformed into part of the luteal cells after ovulation to participate in the regulation of hormone secretion [5]. The process of follicle development is very complex, involving the interaction of multiple biological processes, including hormone regulation, growth factors, signaling, and cell differentiation. Exploring the molecular mechanism of follicular atresia is one of the key points in genetic breeding. Porcine granulosa cells (GCs) isolated from antral follicles represent an ideal and translationally relevant model for investigating follicular atresia. Their advantages include the ease of obtaining ovarian samples from slaughterhouses for GC extraction, ease of primary culture and experimental manipulation, and similar physiological characteristics to human GCs in terms of follicular development and endocrine responses [6].

Follicular atresia is a growth and development process in which the follicle does not mature and ovulate normally and eventually becomes degraded and absorbed [7]. During this process, both the oocyte in the follicle and the surrounding follicular cells also undergo a series of changes. Apoptosis of ovarian granulosa cells is an important cause of follicular atresia. During the development and maturation of follicles, follicular cells (mainly granular cells and membrane cells) around oocytes play an important role in the regulation. These cells secrete a variety of hormones and growth factors that affect oocyte growth and development [8]. Granulosa cells are one of the key cell types in follicle development and maturation. The atresia is closely related to the excessive apoptosis of granulosa cells during follicular atresia, especially during the development of antral follicles to mature follicles. Studies have shown that iron content in granulosa cells is an important regulatory factor during follicular development [9]. Iron metabolism imbalance may lead to impaired cellular antioxidant defense function and fatal accumulation of lipid peroxides, and, ultimately, induce granulosa cell apoptosis, leading to follicular atresia [10,11].

Ferroptosis is a novel form of cell death involving the app-dependent damage of membrane lipids. The metabolic pathways of iron, lipids, and amino acids control the sensitivity of cells to ferroptosis [12]. The process of ferroptosis is characterized by the induction of lipid peroxide accumulation dependent on iron ions and reactive oxygen species [13]. *GPX4* is a glutathione peroxidase in mammals, which catalyzes the reduction of phospholipid hydroperoxides to the corresponding phospholipid alcohols and triggers redox-regulated cell death [14]. Initially, a large number of studies focused on the decrease in *GPX4* activity, leading to the depletion of glutathione. The lipid peroxides cannot be metabolized through the glutathione reductase reaction catalyzed by GPX, resulting in Fe^2+^ oxidation of lipids and the production of reactive oxygen species, which ultimately promote the occurrence of ferroptosis [15]. In 1997, it was found that cellular iron uptake was mainly mediated by the binding of serum transferrin to the transferrin receptor and ferritin endocytosis [16]. In 2016, Wen et al. found that autophagy causes ferroptosis by degrading ferritin in fibroblasts and cancer cells, and ferritin degradation increases the intracellular pool of unstable iron [17]. Since most ROS production is iron-catalyzed, ROS production triggers a lipid peroxidation reaction, leading to the occurrence of ferroptosis in cells [18].

Melatonin was identified 60 years ago as a neurohormone secreted by the pineal gland in the superior thalamus. It is involved in the regulation of various metabolic, immune, reproductive, and other important processes in coordination with the neuroendocrine network system [19]. In mammals, the concentration of melatonin in follicular fluid is significantly higher than that in blood, and its content shows a 24 h rhythm, and the concentration of melatonin continues to increase near the time of ovulation [20]. Intra-follicular oocytes, granulosa cells, and cumulus cells all have the ability to secrete melatonin [21]. Melatonin in the ovary is related to progesterone produced by granulosa luteal cells after ovulation. Different from other tissues, the ovary does not discharge melatonin into the systemic blood, and the melatonin produced by the ovary can protect the cells in the ovary from oxidative stress damage to regulate the microenvironment of the follicle [22]. In this study, we investigated the effect of melatonin on ferroptosis-induced programmed cell death (PCD) of ovarian granulosa cells by rescuing ROS activation in the follicular microenvironment.

## 2. Materials and Methods

### 2.1. Follicle Collection and Initial Processing

#### 2.1.1. Sample Acquisition and Handling

Porcine follicles in this study were collected from a slaughterhouse in Chengdu, Sichuan Province, and transported to the laboratory in sterile, pre-warmed (38 °C) physiological saline within 1 h after slaughter. The ovaries were stored in sterile, preheated (38 °C) saline for subsequent experiments.

#### 2.1.2. Preparation of Transcriptome Sequencing Samples

Upon arrival at the laboratory, intact follicles were immediately dissected (stripped) from the ovaries for RNA-seq sample preparation. After the connective tissue was stripped clean, the follicles were drained of medium on the surface using filter paper, cleaned once with PBS (containing 1% penicillin/streptomycin mixed solution), the surface moisture was drained using filter paper, and the follicles were frozen in liquid nitrogen and placed in a pre-cooled cryostorage tube. After collecting samples, the cryostorage tube was stored in an ultra-low temperature refrigerator at −80 degrees Celsius.

### 2.2. Reagent Sources and Ingredients and Preparation of Commonly Used Reagents

#### 2.2.1. Reagent Sources and Ingredients

RNA isoPLUS was purchased from Takara Bio (Takara Biotech, Dalian, China). AceQ^®^ qPCR SYBR Green Master Mix (High ROX Premixed) and HiScript III RT SuperMix for qPCR (+gDNA wiper) were purchased from Vazyme Biotech (Nanjing Vazyme Biotech Co., Ltd., Nanjing, China). Primers were synthesized by Qingke Biotechnology (Chengdu, China). Physiological saline, anhydrous ethanol (AR grade), 75% ethanol (AR grade), 4% neutral paraformaldehyde fixative, DEPC water were purchased from Haoboyou (Sichuan Agricultural University, Chengdu, China). 1× PBS was purchased from Sevier Bio (Wuhan Sevier Biotechnology Co., Ltd., Wuhan, China). The penicillin/streptomycin mixed solution was purchased from Solebold (Beijing Solebold Technology Co., Ltd., Beijing, China). The DME/F12 culture medium was purchased from Meilun Bio (Dalian Meilun Biotechnology Co., Ltd., Dalian, China). Erastin (5 mg/box) was purchased from Shanghai Yuanye Biotechnology Co., Ltd. (Shanghai, China); DMSO was purchased from Beijing Solebao Technology Co., Ltd. (Beijing, China). The reduced GSH detection kit (E-BC-K030-S), MDA detection kit (E-BC-K025-S), and Fe^2+^ detection kit (E-BC-K881-M) were purchased from Elabscience Biotechnology Co., Ltd. (Wuhan, China). (-)-DHMEQ (Synonyms: Dehydroxymethylepoxyquinomicin), NS-398 (HY-13913), and Luzindole (Synonyms: N-0774) were purchased from MedChemExpress (Monmouth Junction, NJ, USA). CCK-8 reagent was purchased from Beijing Zhuangmeng Biotechnology Co., Ltd. (Beijing, China); EdU-567 kit was purchased from RiboBio Co., Ltd. (Guangzhou, China); JC-1 kit (C2003S), rapid blocking solution, primary antibody diluent, secondary antibody diluent, protease inhibitors, phosphatase inhibitors and ECL color development solution were purchased from Beyotime Biotechnology Co., Ltd. (Haimen, China). The MitoTracker (MitoTracker™ Red CMXRos-M7512) reagent was purchased from Thermo Fisher Scientific (Shanghai, China); C11 BODIPY581/591 lipid peroxidation probe (RM02821) was purchased from Abotek Biotechnology Co., Ltd. (Guangzhou, China). The ROS reagent and BL141A penicillin/streptomycin/gentamicin mixed solution (100× triple antibody) were purchased from Biosharp Life Sciences (Hefei, China). Antibodies were purchased from Beijing Biosun Biotechnology Co., Ltd. (Beijing, China) as follows: p-IκBα (bsm-52169R, 1:1000); p65 (bs-20160R, 1:1000); p-p65 (bs-23303R, 1:500); GPX4 (bs-3884R, 1:500). Melatonin obtained from Shanghai Yuanye Biotechnology Co., Ltd. (Shanghai, China).

#### 2.2.2. Preparation of Commonly Used Reagents

① Erastin reagent was diluted to 10 mM in DMSO. Since this reagent is difficult to dissolve, ultrasonication is required for dissolution. Therefore, it should be prepared immediately before use and diluted 1:1000 in culture medium.

② -(-) DHMEQ: A total of 0.5 mg of powder was dissolved in 100 μL of DMSO to prepare a 19.14 mM stock solution. After ultrasonication, the solution was diluted 1:1000 in culture medium before use.

③ NS-398: A total of 3.149 mg of powder was dissolved in 1 mL of DMSO. After ultrasonication, the solution was diluted 1:1000 in culture medium before use.

④ PTGS2 interfering chain

The interfering chain of PTGS2 was synthesized by RiboBio Co., Ltd. (Guangzhou, China). The specific sequence is shown in Table 1:

⑤ Melatonin: Melatonin was diluted to a 10 μM stock solution (dissolved in anhydrous ethanol). The stock solution was diluted 1:100 to 10^−2^ μM. Then, a 1:1000 dilution was added to the cell culture medium to treat the cells. The final melatonin concentration in the culture medium was 10^−5^ μM. The melatonin solution was stored in the dark.

### 2.3. Isolation and Culture of Primary Ovarian Granule Cells

Ovaries collected as described in Section 2.1.1 were used for follicle dissection. Follicles with good transparency and moderate blood vessel distribution in the size of 4–6 mm were selected after pretreatment, and the follicular fluid was sucked with a 16-gauge needle and collected into a 15 mL centrifugal tube. After collection, the follicular fluid was filtered through a 400-mesh (38 μm) cell sieve, and the filtered fluid was 300 g/5 min. After rinsing with DME/F12 medium, the above steps were repeated once, and the precipitated cell masses were rinsed with 20% FBS medium containing 1% triple antibody, and cell density was calculated with a drop on the cell counting plate. Granular cells were cultured with 20% FBS medium containing 1% triple antibody and transferred to the incubator, and the culture medium was changed after cell adhesion was observed the next day. When the cell density reaches about 80% and the passage begins, it is generally necessary to re-isolate the primary cells after 203 generations of passage. The culture medium in the culture bottle was discarded, 0.25% pancreatic enzyme was added, and the cells were incubated in the incubator for 1 min, then removed, and placed under a microscope to observe whether the cell morphology was completely digested. After adding 20% FBS medium containing 1% triple antibody to the cell bottle, digestion was terminated, and the cell suspension in the cell culture bottle was gently blown with a Pap straw. The cell suspension was inhaled into a 15 mL centrifuge tube, centrifuged at 2500 g for 3 min, then the supernatant was discarded, fresh cell medium was added, then blown well (be careful not to produce bubbles), and the cell suspension was divided into two equal parts and transferred to the cell bottle and placed in the incubator. After cell adhesion, the purity of primary ovarian granulosa cells was determined by FSHR immunofluorescence staining.

### 2.4. Dissection, Culture, and Treatment of Porcine Follicles

Ovaries collected as described in Section 2.1.1 were used. After the blood stains were cleaned with 38 ± 1 °C normal saline, the sow ovaries were placed in 1× PBS containing 1% green streptomycin mixed solution, and then placed in a vehicle refrigerator preheated to 38.6 °C and brought back to the laboratory for follow-up treatment. After taking the ovaries back to the laboratory, the ovaries were washed twice with clean 1× PBS, washed with 75% ethanol (AR) grade for 15 s, and then washed twice with 1× PBS to begin the follicle stripping. A scalpel was used to cut the cortical part of the surface of the ovary. After tearing the cortical part with eye tweezers, the follicle was separated from the surrounding adhesive tissue with tweezers and gently moved from the root (medullary part) to the left and right, and then slowly pulled out, finally peeling off the complete follicle. After the follicles were cleaned twice with DME/F12 medium containing 1% penicillin/streptomycin mixed solution, they were inserted into a culture dish with DME/F12 medium containing 1% penicillin/streptomycin mixed solution using high-temperature sterilized eye tweezers and then cultured in a cell incubator containing 37 °C and 5% CO_2_.

The follicles were gently grasped with ophthalmic forceps and placed in a cell culture plate, and an appropriate amount of culture medium was added. A total of 1 μL of DMSO was added to each milliliter of culture medium in the control group, and 1 μL of erastin (10 μm concentration) was added to each milliliter of culture medium in the experimental group for 48 h.

### 2.5. Immunofluorescence Staining

The cell climbing tablets were placed in 6-well plates, cell suspension was added, and the cells were treated accordingly after adhesion. After fixation with paraformaldehyde, the cells were cleaned with PBS for 1 to 2 times for 5 min each time, and the cells were treated with puncture solution (3% concentration) for 30 min. After that, the cells were cleaned 1 to 2 times, and 3% BSA was added to the cell plates to evenly cover the tissues. After that, the cells remained closed at room temperature for 30 min. The first antibody diluted with the first antibody diluent was added to the cell pore plate, and the cell culture plate was placed flat in a wet box at 4 °C and incubated overnight. The cell pore plate was placed on the decolorizing shaking table and washed 3 times, 5 min each time. Corresponding secondary antibodies were added and incubated at room temperature for 50 min. DAPI restaining nuclei: The crawling tablets were placed in PBS (PH 7.4) and washed by shaking on the decolorizing shaker 3 times, 5 min each time. After the slices were slightly dried, DAPI dye was added to the circle and incubated for 10 min at room temperature away from light. The climbing tablets were placed in PBS (PH 7.4) and washed by shaking on the decolorizing table 3 times, 5 min each time. The slide was slightly dried and sealed with an anti-fluorescence quenching sealant. Finally, the image was collected and the exported file was scanned, and the CS software (Ver. 2.3) was used for observation.

### 2.6. Cell Cycle Detection

After the treatment, 1 × 10^6^ cells were collected, the supernatant was discarded after centrifugation, the cell mass was rinsed with 75% ethanol, and the cells were fixed overnight at −20 °C. DNA staining solution in the amount of 1 mL was added, and the staining was mixed with vortex staining for 5–10 s. Then, the solution was incubated at room temperature for 30 min away from light. The minimum loading speed was selected and detected by flow cytometry.

### 2.7. Criteria for Determining the Degree of Follicular Atresia

The peeled fresh follicles were absorbed using filter paper to remove the residual PBS containing 1% green streptomycin mixed solution from the outside of the follicles, and the follicles of 4–5 mm were selected for subsequent experimental treatment by measuring the diameter of the follicles with vernier calipers. The blood vessels on the surface of healthy follicles are abundant, transparent, and pink in color. The primary atretic follicle shows a small number of blood vessels, which are pale pink in color and poor in transparency. There is no blood vessel distribution on the surface of the atretic follicle, the color is milky yellow, and the internal follicle is cloudy. The criteria are listed in Table 2.

### 2.8. Real-Time PCR

RNA was extracted by RNAiso PLUS method. RNA was reverse-transcribed using HiScript III RT SuperMix for qPCR (+gDNA wiper). The 2× AceQ qPCR SYBR Green Master Mix (High ROX Premixed) was used for fluorescence quantification. Primers are listed in Table 3. Reaction steps: Place the fluorescent quantitative sample plate in the groove inside the machine, close the machine lid, and set the amplification program: ① pre-denaturation at 94 °C for 300 s, ② denaturation at 94 °C for 30 s, ③ annealing at 60 °C for 30 s, ④ extension at 72 °C for 30 s, wherein ②–④ are cycled 40 times, and then the temperature is lowered to 4 °C to end the amplification detection.

### 2.9. Detection of Indicators

#### 2.9.1. ROS Detection Assay

The probe was diluted with 1:1000 serum-free medium and added to the cell culture plate. The cell plate was incubated in the incubator for 30 min, and the medium was replaced with fresh medium.

#### 2.9.2. Mitochondrial Membrane Potential (ΔΨm) Assay

A total of 5 microliters of JC-1 concentrated working liquid was added to serum-free medium, diluted 1:1 with JC-1 buffer, mixed well, and kept away from light. To the six-well cell plate, 1 mL fresh media and 1mL JC-1 working solution were added, and the cell culture plate was incubated in an incubator for 40 min. After that, the cells were washed with JC-1 buffer solution once, and the fluorescence excitation of the cells was observed under an inverted fluorescence microscope.

#### 2.9.3. MitoTracker Staining

A total of 50 μg of MitoTracker powder was taken from one tube and 94.06 μL of DMSO was added to prepare a 1 mM stock solution. The stock solution was diluted to 500 nM with serum-free medium and added to the cell culture plate. It was incubated for 30 min, washed twice with preheated 1× PBS, and the cells were fixed with 4% paraformaldehyde for 15 min before observing under a fluorescence microscope.

#### 2.9.4. CCK-8

CCK-8 reagent was added to a 96-well cell culture plate, 10 μL CCK-8 reagent was added to 100 μL medium. After that, the cell culture plate was incubated in the incubator for 2 h, and the absorbance at 450 nm was determined by enzyme labeling.

#### 2.9.5. EDU

The EdU solution (reagent A) was diluted with cell complete culture medium at a 1000:1 ratio to prepare an appropriate EdU medium of 50 μM. A total of 100 μL 50 μM EdU medium was added to each well, incubated for 2 h, and the medium was discarded. Then, 1× PBS was added, and the dye was washed 1–2 times. After fixing the cells with 50 μL of 4% paraformaldehyde at 4 °C overnight, 50 μL of 2 mg/mL glycine was added and incubated for 5 min at room temperature. The supernatant was discarded, then 1× PBS was added and washed 1–2 times. The cells were then permeabilized with 150 μL of 0.5% Triton X-100 and incubated with 100 μL of the Apollo^®^ staining reaction cocktail in the dark for 60 min. Then, the dyeing reaction solution was discarded. Reagent F was diluted in a ratio of 100:1 in deionized water, and an appropriate amount of Hoechst33342 working liquid was prepared and stored away from light. A total of 100 μL Hoechst33342 working liquid was added to each well and incubated in a decolor shaker at the dark room temperature for 30 min. The staining reaction liquid was discarded, 1× PBS was added, washed twice, and observed under a microscope.

#### 2.9.6. Reduced Glutathione (GSH) Assay

Preparation of supernatant: 1. Take 0.1 mL sample to be tested, add 0.1 mL reagent 1, mix well, centrifuge at 4500× *g* for 10 min, and take supernatant to be tested (if the supernatant contains part of the precipitate, transfer the supernatant into a new EP tube and centrifuge again). 2. Add 25 μL reagent 3 to the standard, determination, and determination blank holes. Determination of blank hole: add 100 μL reagent 1; standard hole: add 100 μL GSH standard solution with different concentrations; test hole: add 100 μL supernatant. 3. Add 100 μL reagent 2 to each hole in step 2. 4. Vibrate the plate of the enzyme marker for 1 min, let it stand for 5 min, and measure the OD value with the enzyme marker at 405 nm.

#### 2.9.7. MDA Detection Method

Before detection, the reagent in the kit was balanced to room temperature. 1. Blank tube: Take a milliliter of anhydrous ethanol and add it to a 10 mL glass test tube; standard tube: Take 10 nmol/mL standard product and add it to a 15 mL centrifuge tube; test tube and control: Take 0.1 mL of each sample to be tested and add it to a 10 mL glass test tube. 2. Add 0.1 mL reagent 1 to each tube in step 1. 3. Add 3.0 mL of reagent 2 application solution to each tube in Step 2. 4. Add 1.0 mL reagent 3 application solution to the blank, standard, and measuring tubes in step 3, and add 1.0 mL 50% glacial acetic acid to the center. 5. After thoroughly mixing, tie the mouth of the test tube tightly with plastic wrap, tie a small hole in the plastic wrap, and bathe in water above 95 °C for 40 min. 6. After removal, cool to room temperature with water and centrifuge for 10 min at 3100× *g*. 7. Take 3 mL of supernatant liquid and add it to a 1 cm optical diameter quartz colorimetric dish with a wavelength of 532 nm, then adjust the double steaming water to zero to measure its absorbance.

#### 2.9.8. Ferrous Ion Detection

Standard hole: 1. Take 200 μL standard product with different concentrations and add it to the corresponding hole of the enzyme label plate, respectively. Assay hole: Take 200 μL sample and add it to the corresponding hole of the enzyme label plate. 2. Add 100 μL reagent 2 to each hole in step 1. 3. Mix well and incubate at 37 °C for 10 min. 4. Measure OD values of each hole at 593 nm of the enzyme marker.

#### 2.9.9. BODIPT581/591 Labeling Experiment

Dissolve 1 mg of BODIPY 581/591 in 198.2 μL of DMSO to prepare a 10 mM stock solution. After the cells have reached the appropriate density, add an appropriate volume of C11 BODIPY 581/591 to a final concentration of 10 μM and incubate for 1 h. Wash the cells twice with PBS to remove excess dye. Then, trypsinize the cells and resuspend them in PBS containing 5% FBS. Using a flow cytometer, measure the signal corresponding to 505–550 nm in the FL1(FITC) channel using a 488 nm laser excitation. Measure the signal above 580 nm in the FL2(PE) channel using a 565 nm laser excitation. Construct histograms based on the signals in the FL1 and FL2 channels.

### 2.10. Data Statistics and Analysis

Transcriptome sequencing data were analyzed; firstly, follicle RNA was extracted, and then RNA quality assessment was performed. The degradation degree of RNA was analyzed by agarose gel electrophoresis, and the purity of RNA was detected by Nanodrop (OD260/280 ratio), Qubit for accurate quantification of RNA concentration, and Agilent 2100 for accurate detection of RNA integrity. The full-length transcriptome library was constructed using the Oligo(dT) magnetic bead enrichment method, and the library was sequenced on the computer. After the sequencing, the original data were de-splined, and low-quality reads were removed, and the data were analyzed by GO and KEGG. The bubble chart and clustering heat maps are in dior biological information cloud platform https://www.omicshare.com/tools/ (accessed on 1 August 2024).

The qRT-PCR data were analyzed using the 2^−ΔΔCt^ method (Livak method), and the biochemical data and the quantitative data of real-time fluorescence PCR were analyzed and plotted using Prism 9.0. Finally, the biochemical data and the quantitative data of real-time fluorescence quantitative PCR were analyzed in Prism 9.0, and the pictures were drawn. The other data of this study were analyzed using Prism 9.0. Correlation analysis, one-way analysis of variance (ANOVA), and multiple comparisons between groups were performed using Prism 9.0. All data in this study were analyzed using the mean value. All the data of the experiment were expressed in the form of mean (Mean) ± standard deviation (SD), and * represents a significant difference with *p* < 0.05 *, *p* < 0.01 **, *p* < 0.001 ***, *p* < 0.0001 ****.

## 3. Results

### 3.1. Different Levels of Ferroptosis in Healthy and Atretic Follicles of Pigs

**Figure 1:**
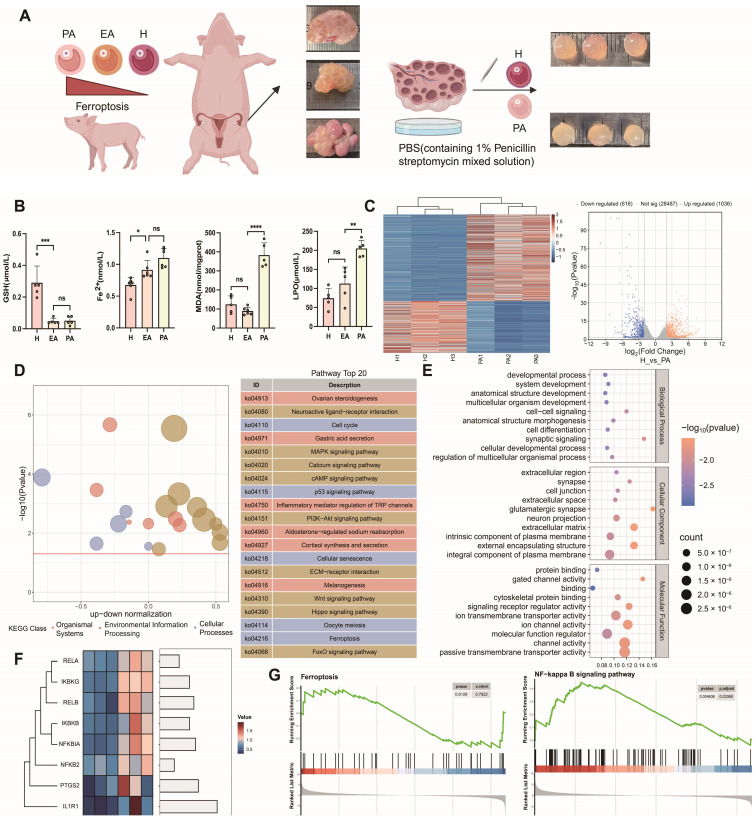
Transcriptome sequencing analysis of healthy and atretic follicles. (**A**) Healthy follicles (H), primary atretic follicle (EA), and atretic follicles (PA) were collected from different ovaries of pigs for transcriptome sequencing. In the schematic diagram of ovarian tissue, arrows indicate follicles, red arrows: healthy follicles, black arrows: atretic follicles. (**B**) Differences in the content of ferroptosis markers among healthy follicles, primary atresia follicles, and atresia follicles. (**C**) Volcanic and heat maps of differential genes in healthy follicles and atretic follicles. (**D**) Differential gene GO enrichment analysis of healthy follicles and atretic follicles. (**E**) Differential gene KEGG enrichment analysis of healthy follicles and atretic follicles. (**F**) Differential expression of *NF-κB*/*PTGS2-related* genes by transcriptome sequencing. (**G**) *NF-κB* and KEGG gene set enrichment analysis of the ferroptosis pathway. Significance: *, *p* < 0.05; **, *p* < 0.01; ***, *p* < 0.001; ****, *p* < 0.0001.

Freshly obtained follicle samples were used for testing and sequencing (Figure 1A). We detected healthy follicles, primary atretic follicles (EA), ferroptosis markers in atretic follicles (PA), GSH, Fe^2+^, MDA, LPO, downregulated GSH content in atretic follicles, and upregulated expression of Fe^2+^, MDA, and LPO (Figure 1B). RNA-seq results showed that there were a total of 1654 differentially expressed genes in healthy and atretic follicles (Figure 1C), GO (Figure 1D), and KEGG (Figure 1E). The analysis results also showed that differential gene function was enriched in the cell cycle, *MAPK*, *PI3K-AKT*, *P53*, *TGF-β*, and Hippo signaling pathways. *PTGS2* is a marker gene for ferroptosis, and both *NF-κB*/*PTGS2* genes are upregulated in atretic follicles. Some *NF-κB* signaling pathway genes were screened out from differential genes by transcriptome sequencing, and key transcription factors of this pathway were significantly upregulated in atretic follicles (Figure 1F). *NF-κB* signaling was significantly enriched in differential genes between atretic follicles and healthy follicles, and some genes of the ferroptosis pathway were associated with follicular atretic (Figure 1G).

### 3.2. Ferroptosis Inhibits Granulosa Cell Proliferation and Induces Oxidative Stress

**Figure 2:**
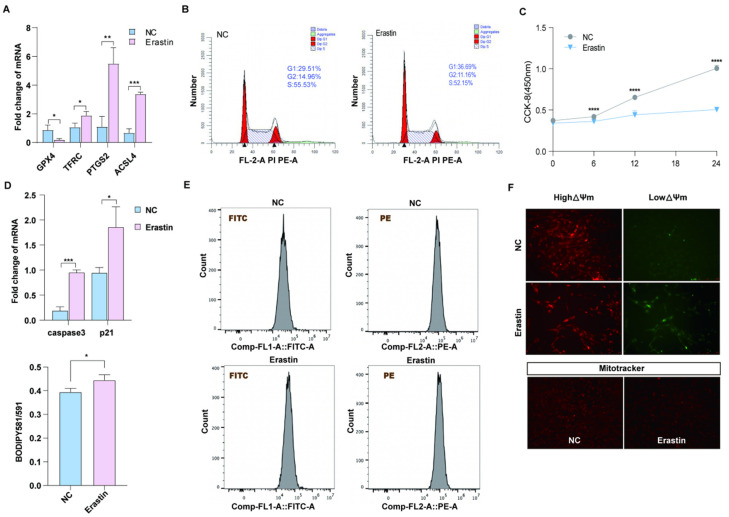
The effect of ferroptosis on primary porcine ovarian granulosa cells. (**A**) qRT-PCR was used to detect the expression of ferroptosis marker genes. *p* < 0.05 *, *p* < 0.01 **, *p* < 0.001 ***. (**B**) Flow cytometry results in the NC and erastin-treated groups. (**C**) CCK-8 was used to detect cell proliferation after NC and erastin treatment. (**D**) qRT-PCR was used to detect the expression of apoptosis marker genes. (**E**) JC-1 was used to detect the changes in mitochondrial membrane potential in the NC and erastin treatment groups. (**F**) MitoTracker activity in NC and erastin treatment groups. Significance: *, *p* < 0.05; **, *p* < 0.01; ***, *p* < 0.001; ****, *p* < 0.0001.

Previous studies have shown that GPX4, TFRC, PTGS2, and ACSL4 are marker genes for ferroptosis [23]. We treated porcine primary ovarian granulosa cells with 10 μM erastin and detected the upregulation of ferroptosis marker genes TFRC, PTGS2, and ACSL4 by qRT-PCR, while GPX4 was downregulated (Figure 2A). In order to explore the effect of ferroptosis on ovarian granulosa cell proliferation and its regulatory mechanism, the cell cycle of the control group and the treatment group was detected by flow cytometry (Figure 2B). The proportion of cells in G2 + S phase in the erastin group was smaller than that in the control group. CCK-8 results showed that the cell proliferation ability of the erastin-treated group was significantly decreased after 12 h of treatment (Figure 2C). The qRT-PCR results also showed that the expression levels of cell apoptosis marker genes caspase3 and p21 were upregulated in the erastin group (Figure 2D). Using flow cytometry Bodipy 581/591 probe and detecting erastin granulosa cells in the treatment group and control group of lipid peroxidation, we obtained results showing that compared with the control group, erastin lipid peroxidation rate rose significantly in the treatment group (Figure 2E). Since oxidative stress is closely related to mitochondrial damage, JC-1 and MitoTracker fluorescence staining were used to detect mitochondrial membrane potential and mitochondrial activity in the erastin group and the control group. The results showed that mitochondrial membrane potential was reduced and mitochondrial activity was damaged in the erastin-treated group (Figure 2F).

### 3.3. The NF-κB in the Nuclear Inhibitors Inhibits Granular Cell Death and Oxidative Stress and Promotes Proliferation

**Figure 3:**
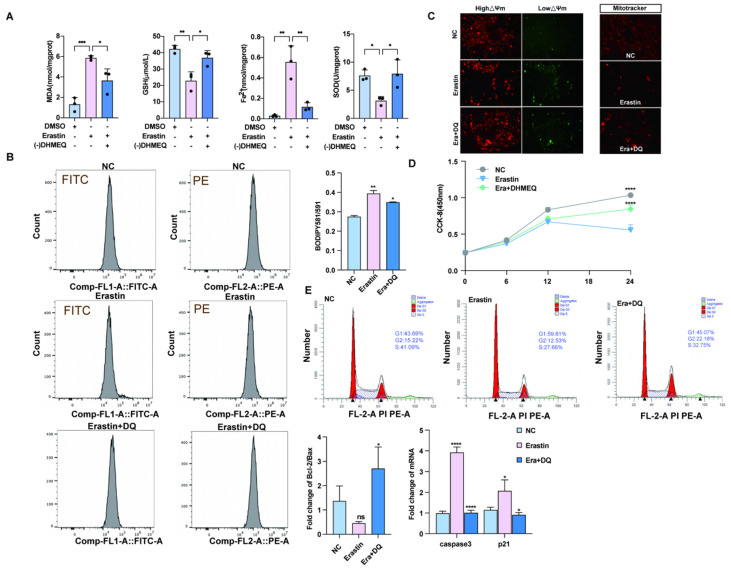
Blocking the nuclear import of *NF-κB* can rescue the effect of ferroptosis on primary porcine ovarian granulosa cells. (**A**) Ferroptosis markers after NC, erastin, erastin + -(-) DHMEQ treatment. (**B**) The ratio of lipid peroxidation after NC, erastin, erastin + -(-) DHMEQ treatment on ovarian granulosa cells. (**C**) JC-1 was used to detect the changes in mitochondrial membrane potential after NC, erastin, and erastin + -(-) DHMEQ treatment. MitoTracker activity after NC, erastin, erastin + -(-) DHMEQ treatment. (**D**) CCK-8 was used to detect the effects of NC, erastin, and erastin + -(-) DHMEQ on the proliferation of ovarian granulosa cells. (**E**) qRT-PCR was used to detect the effects of NC, erastin, erastin + -(-) DHMEQ treatment on *Bcl2/BAX* regulatory axis and apoptosis marker genes in ovarian granulosa cells. NC, erastin, erastin + -(-) DHMEQ after processing by flow cytometry to detect cell proliferation cycle. Significance: *, *p* < 0.05; **, *p* < 0.01; ***, *p* < 0.001; ****, *p* < 0.0001.

To investigate whether the *NF-κB* preferred pathway plays a regulatory role in ferroptosis agonist-induced ovarian granulosa cell death, we treated ovarian granulosa cells with a specific *NF-κB* pathway inhibitor -(-) DHMEQ. The levels of ferrodeath markers MDA, GSH, SOD, and Fe^2+^ were measured in the rescue group supplemented with -(-) DHMEQ, and the results showed that the contents of MDA, GSH, SOD, and Fe^2+^ returned to normal levels after the addition of -(-) DHMEQ (Figure 3A). In addition, flow cytometry was used to detect lipid peroxidation among different groups. We found that lipid peroxidation level was significantly upregulated after erastin treatment, and addition of -(-) DHMEQ could effectively rescue this phenomenon (Figure 3B). In addition, by JC–1-(-), after processing the low-potential green fluorescent DHMEQ rescue group, MitoTracker staining was reduced, and fluorescence quantitative results show red fluorescence enhancement DHMEQ rescue group, with mitochondrial function restoration (Figure 3C). To further explore whether -(-) DHMEQ could affect granulosa cell proliferation, erastin-inhibited cell proliferation after 12 h of cell treatment was rescued by the addition of -(-) DHMEQ. The *p21* quantitative results tended to be consistent with the CCK-8 results (Figure 3D). The results of cell cycle detection by flow cytometry showed that the low proportion of S + G2 phase cells caused by erastin could be restored after the addition of -(-) DHMEQ (Figure 3E).

### 3.4. Inhibition of PTGS2 Promotes the Proliferation of Granulosa Cells and Alleviates Ferroptosis

**Figure 4:**
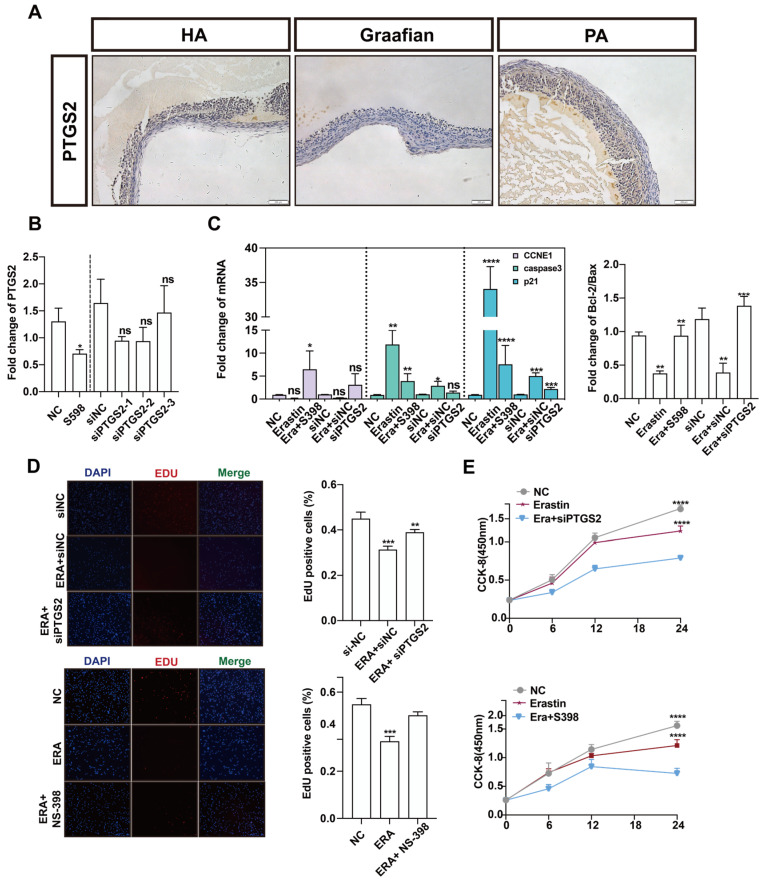
The effect of *PTGS2* inhibition on the proliferation of primary porcine ovarian granulosa cells. (**A**) The expression of *PTGS2* in healthy follicles (HA), mature follicles (Graafian follicle), and atretic follicles (PA) was detected by immunohistochemistry (200 um ruler in the lower right corner). (**B**) qRT-PCR was used to detect the inhibition efficiency of the *PTGS2* gene by NC, NS-398, and si*PTGS2* treatment. (**C**) qRT-PCR was used to detect the effects of NC, erastin + NS-398, and erastin + si*PTGS2* on the *Bcl2/BAX* regulatory axis, cell proliferation, and apoptosis marker genes in ovarian granulosa cells. Ovarian granulosa cells were treated. (**D**) EDU immunofluorescence observation added staining pictures of the E group, E + NS398 group, siNC group, E + siNC group, E + si*PTGS2* group, and the control group. (**E**) The results of the cck assay at 6 h, 12 h, 18 h, and 24 h of cells added to the E group, E + si*PTGS2* group, E + NS398 group, and the control group. Significance: *, *p* < 0.05; **, *p* < 0.01; ***, *p* < 0.001; ****, *p* < 0.0001.

*PTGS2* is one of the important marker genes in the process of ferroptosis. Many studies have reported that the *NF-κB*/*PTGS2* axis promotes inflammation and regulates apoptosis. We explored the role of *PTGS2* in the process of follicular atresia. Immunohistochemistry was performed on healthy and atretic follicles and mature follicles. Compared with healthy follicles, *PTGS2* was low in atretic follicles and very low in mature follicles (Figure 4A). These results suggest that *PTGS2* may play a key regulatory role in the dynamic changes in follicular development and follicular atresia. *PTGS2* was specifically inhibited by NS-398, and the expression of *PTGS2* in the NS-398 treatment group decreased by about two times compared with the control group. At the same time, the interference efficiency of si*PTGS2* was up to 70%. Subsequent experiments were performed with siPTGST-1 (Figure 4B). To explore the effect of *PTGS2* expression reduction and ferroptosis on the proliferation of ovarian granulosa cells, NS-398 and siPTGST-1 were added after erastin treatment, respectively. The expression of proliferation marker CCNDE1 was upregulated after NS-398 was added to the erastin group. The expression of cell apoptosis marker genes *caspase3* and *p21* was downregulated, and *CCNE1* was significantly upregulated after adding siPTGST-1. *Bcl-2/bax* had an upward trend after adding NS-398 or siPTGST-1 (Figure 4C). EdU experimental results showed that, in both the NS-398 and siPTGS2 groups, cell proliferation quantity was far greater than that in the erastin groups (Figure 4D). The CCK-8 experimental results presented the same trend (Figure 4E). Together, these results show that inhibition of PTGS2 can rescue the cell proliferation inhibition phenomenon caused by erastin-induced ferroptosis.

**Figure 5:**
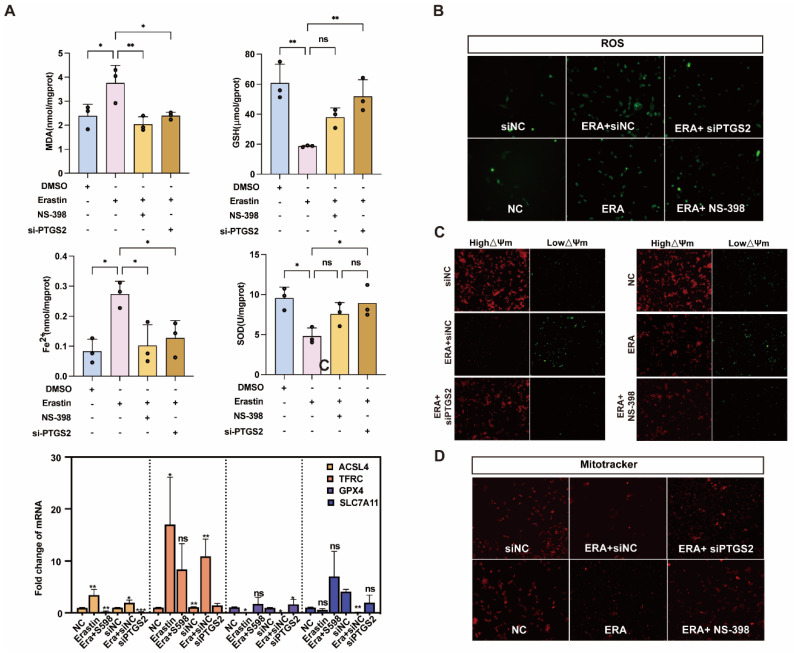
Inhibition of *PTGS2* expression rescued ferroptosis and mitochondrial damage in primary porcine granulosa cells. (**A**) Detection of ferroptosis markers in ovarian granulosa cells treated with NC, erastin + NS-398, and erastin + si*PTGS2*. qRT-PCR was used to detect the inhibition efficiency of NC, erastin, erastin + NS-398, and erastin + si*PTGS2* in ferroptosis marker genes. (**B**) The levels of reactive oxygen species (ROS) in ovarian granulosa cells treated with NC, erastin + NS-398, and erastin + si*PTGS2* were detected. (**C**) The mitochondrial activity of ovarian granulosa cells treated with NC, erastin + NS-398, and erastin + si*PTGS2* was detected by MitoTracker. (**D**) The mitochondrial membrane potential of ovarian granulosa cells was detected by NC, erastin + NS-398, and erastin + si*PTGS2* treatment. Significance: *, *p* < 0.05; **, *p* < 0.01; ***, *p* < 0.001.

We further explored the relationship between *PTGS2* and ferroptosis of ovarian granulosa cells. The erastin-induced ferroptosis model was also rescued with NS-398 or siPTGST-1. The contents of MDA, GSH, Fe^2+,^ and SOD in the NS-398 or siPTGST-1 group were rescued compared with the erastin treatment group. qRT-PCR results showed that *ACSL4,* which promoted ferroptosis after adding NS-398 or siPTGST-1, had a statistically significant difference compared with the erastin treatment group. Compared with the erastin treatment group, the expression of TFRC was downregulated, the ferroptosis marker A gene *GPX4* was inhibited, and the expression of *SLC7A11* was significantly upregulated (Figure 5A). Because ferroptosis can cause mitochondrial pyknosis, mitochondrial membrane potential change, and reactive oxygen species accumulation, we detected mitochondrial function, mitochondrial membrane potential, and reactive oxygen species by MitoTracker, JC-1, and ROS fluorescence staining in NC, erastin, erastin + NS-398, and erastin + si*PTGS2*, respectively. The results showed that mitochondrial activity was significantly upregulated, mitochondrial membrane potential was restored, and reactive oxygen species (ROS) accumulation was reduced in erastin + NS-398 and erastin + si*PTGS2* groups compared with the erastin treatment group (Figure 5B–D).

### 3.5. Melatonin Rescued Erastin-Induced Ferroptosis

**Figure 6:**
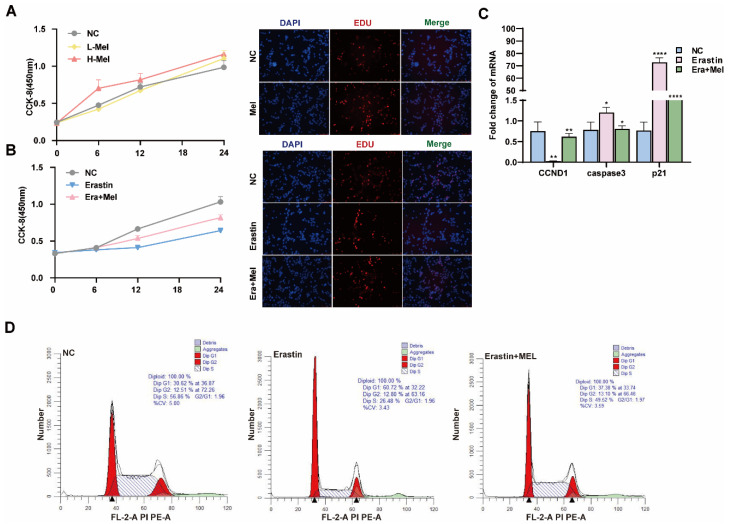
Melatonin rescues the inhibitory effect of ferroptosis on the proliferation of primary porcine ovarian granulosa cells. (**A**) CCK-8 was used to detect the effect of different concentrations of melatonin on the proliferation of ovarian granulosa cells. The effect of high concentration of Mel on the proliferation of ovarian granulosa cells was detected by EdU. (**B**) CCK-8 and EdU were used to detect the proliferation ability of NC, erastin, and erastin + Mel groups. (**C**) The effects of NC, erastin, and erastin + Mel on cell cycle and apoptosis marker genes were detected by qRT-PCR. (**D**) The effects of NC, erastin, and erastin + Mel on the cell cycle were detected by flow cytometry. Significance: *, *p* < 0.05; **, *p* < 0.01; ****, *p* < 0.0001.

**Figure 7:**
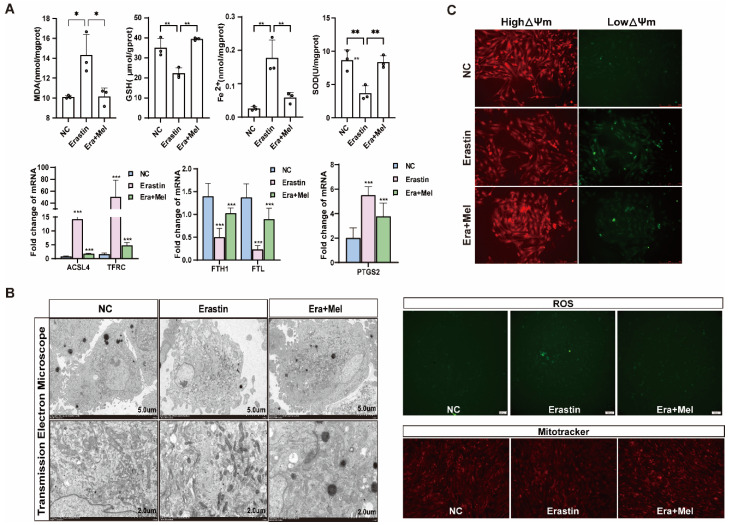
Melatonin rescued ferroptosis and mitochondrial damage in primary porcine ovarian granulosa cells. (**A**) The content of ferroptosis markers in NC, erastin, and erastin + Mel groups was detected. (**B**) The effects of NC, erastin, and erastin + Mel on ferroptosis were detected by transmission electron microscopy. (**C**) The changes in mitochondrial membrane potential in ovarian granulosa cells treated with NC, erastin, and erastin + Mel were detected by MitoTracker. Effects of NC, erastin, and erastin + Mel on ROS in ovarian granulosa cells. MitoTracker staining was used to detect the mitochondrial activity of ovarian granulosa cells treated with NC, erastin, and erastin + Mel. Significance: *, *p* < 0.05; **, *p* < 0.01; ***, *p* < 0.001.

Melatonin is one of the indole heterocyclic compounds, which is one of the hormones secreted by the pineal gland of the brain. It has the functions of regulating circadian rhythm and neuroendocrine immunomodulatory activity, scavenging free radicals, and showing antioxidant ability [24]. In order to explore the molecular mechanism of melatonin to rescue ferroptosis-induced ovarian granulosa cells damage, the effects of high and low concentrations of melatonin on ovarian granulosa cells proliferation were detected by 10^−6^ μM and 10^−5^ μM melatonin. CCK-8 and EdU assays showed that a low concentration of melatonin more significantly promoted the proliferation of ovarian granulosa cells (Figure 6A). We further detected cell proliferation in the control, erastin-treated, and 10^−6^ μM melatonin + erastin groups. The results demonstrated that melatonin promoted the proliferation of granulosa cells even after the addition of erastin (Figure 6B). In addition, the cell proliferation marker gene *CCND1* was significantly upregulated in the melatonin rescue group, and the apoptosis genes *caspase3* and *p21* were significantly downregulated in the melatonin complete group (Figure 6C). Flow cytometry was used to detect the cell cycle of the above three treatments, and it was found that S + G2 phase cells in the erastin group significantly decreased compared with the control group, and increased after the addition of melatonin (Figure 6D). We further examined the effect of melatonin on ferroptosis markers after erastin treatment. MDA and Fe^2+^ were significantly downregulated, while GSH and SOD were significantly upregulated in the rescue group after melatonin addition. FTL inhibited the expression of ferroptosis marker genes (Figure 7A). Using transmission electron microscopy to observe the morphological changes in mitochondria and other cells in the erastin group and erastin + MEL group, we found that pyknotic or swollen mitochondria and the disappearance of mitochondrial cristae occurred in the erastin group. After adding MEL, the pyknotic mitochondria were reduced and a few mitochondrial cristae were clearly outlined (Figure 7B). MitoTracker, JC-1, and ROS fluorescence staining were used to detect mitochondrial activity, mitochondrial membrane potential, and reactive oxygen species (ROS) content in the erastin group and erastin + MEL group, respectively. We found that MEL could effectively rescue mitochondrial activity and restore mitochondrial membrane potential. Figure 7C shows the reduction in reactive oxygen species. The above results suggest that melatonin plays a protective role in ferroptosis-induced cell damage.

### 3.6. Melatonin Receptor Inhibitors Weaken the Ferroptosis-Inhibiting Effect of Melatonin in GCs

**Figure 8:**
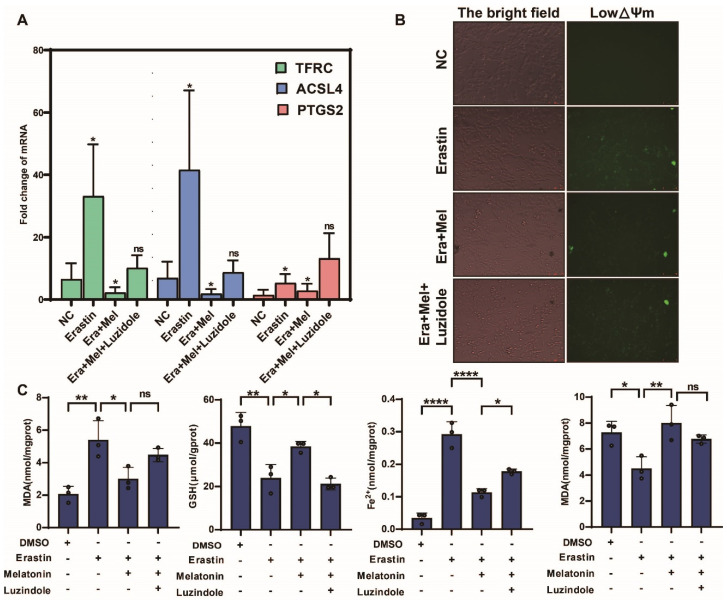
Melatonin receptor inhibitors weaken the ferroptosis-inhibiting effect of melatonin in GCs. (**A**) Detection of ferroptosis marker genes in ovarian granulosa cells treated with NC, erastin, erastin + Mel, and erastin + Mel + luzidole by qRT-PCR. (**B**) Detection of mitochondrial membrane potential changes in ovarian granulosa cells treated with NC, erastin, erastin + Mel, and erastin + Mel + luzidole. (**C**) was used to detect the changes in ferroptosis markers in ovarian granulosa cells treated with NC, erastin, erastin + Mel, and erastin + Mel + luzidole. Significance: *, *p* < 0.05; **, *p* < 0.01; ****, *p* < 0.0001.

In order to further verify the salvage effect of melatonin on ovarian granulosa cells after ferroptosis injury, this study added erastin to ovarian granulosa cells and verified the above function by melatonin and melatonin receptor inhibitor luzidole (10 μg/mL), as shown in Figure 8A. The inhibitory effect of melatonin on the expression of ferroptosis marker gene was significantly weakened after the addition of a melatonin inhibitor, but the inhibitory effect on ferroptosis marker gene was the opposite. The results of JC-1 staining confirmed that the addition of melatonin receptor inhibitors weakened the inhibitory effect of melatonin on ferroptosis and aggravated the changes in mitochondrial transmembrane potential (Figure 8B). Finally, biochemical kits were used to detect the ferroptosis markers MDA, GSH, Fe^2+,^ and SOD. The results showed that the content of the markers promoting ferroptosis in the cells was significantly upregulated after adding melatonin inhibitors, while the content of reduced GSH and SOD was significantly downregulated (Figure 8C).

## 4. Discussion

The transcriptomic analysis revealed significant enrichment of ferroptosis-related pathways and genes in porcine atretic follicles (Figure 1), suggesting a potential role for this novel form of regulated cell death in follicular atresia. Importantly, alongside established ferroptosis markers, the NF-κB signaling pathway and its downstream target PTGS2 were significantly upregulated. This led us to hypothesize that the NF-κB/PTGS2 axis might be a key executor of ferroptosis in granulosa cells. To test this, we utilized porcine ovarian granulosa cells as a translational model [6] and employed erastin to induce ferroptosis. Ferroptosis is a dependent regulatory cell death caused by iron overload and excessive accumulation of reactive oxygen species, dependent on lipid peroxidation [25]. When ferroptosis occurred, the morphological characteristics of the cells were mitochondrial shrinkage, mitochondrial ridge reduction or absence, and mitochondrial membrane density increase. Ferroptosis plays a crucial role in different biological processes. Its bioenergy characteristics are mainly iron accumulation and lipid peroxidation [18]. Our subsequent experiments confirmed that erastin treatment successfully triggered the hallmarks of ferroptosis in GCs, including lipid peroxidation, mitochondrial dysfunction, and, ultimately, cell death (Figure 2, Figure 3A, Figure 5A, and Figure 7A).

Atresia occurs in more than 90% of follicles in mammals, which seriously affects the utilization of reproductive resources [26]. The pig’s reserve of primordial follicles forms in the fetal ovaries, with about 5 million primordial follicles available during puberty. During each estrous cycle under hormonal stimulation, a cohort of primordial follicles is initiated to grow. These follicles may either continue developing to the pre-ovulatory stage or undergo atresia at any point during their development. Most follicles become atretic once they exceed 1 mm in diameter, and the atresia rate increases markedly in antral follicles approximately 3–5 mm in size. Studies on different developmental stages of sinus growing follicles have shown that follicle atresia is caused by apoptosis of granulosa cells GC, which is regulated by the balance of pro-survival factors and pro-apoptotic factors [27]. Early transcriptome analysis of atretic follicles showed that the results of gene cluster analysis were different at different developmental stages. There have been reports of follicular atresia, but the specific mechanism still needs to be elucidated to a large extent. In atretic follicles, glutathione content is decreased while the expression of ferroptosis marker genes is upregulated. To investigate the role and regulatory mechanism of ferroptosis in follicular atresia, this study performed RNA-seq on both healthy and atretic follicles. Potential pathways involved in the regulation of follicular atresia were identified through GO, KEGG, and GSEA enrichment analyses. Using porcine ovarian granulosa cells as a model, we further explored the impact of ferroptosis on ovarian granulosa cell death. Our findings revealed significant enrichment of signaling pathways, including FOXO and PI3K-AKT. Further analysis also highlighted the enrichment of pathways related to inflammatory response and cysteine metabolism, with the latter exhibiting a negative correlation trend. Based on these results, this study further examined the role of the NF-κB/PTGS2 regulatory axis, which is known to govern inflammatory response, in ferroptosis-induced granulosa cell injury.

The key effector of the *NF-κB* signaling pathway is the entry of p65 into the nucleus, which acts as a cellular transcription factor to promote transcription of downstream factors. Complete elimination of the *NF-κB* signaling pathway results in ovarian dysfunction in mice, with mesoovarian sinusoid eggs. The number of bubbles is significantly reduced. However, abnormal activation of the *NF-κB* pathway can lead to overexpression of inflammatory factors [28,29] and eventually lead to ovarian granulosa cell death, resulting in follicular atresia. In this study, immunofluorescence staining was used to detect the induction of ferroptosis in eggs. The nuclear translocation of p65 was significantly increased, and the expression of downstream marker genes was upregulated during the death of nest granulocyte cells, suggesting that the *NF-κB* pathway is abnormally activated in the environment of ferroptosis and inhibited by the addition of melatonin.

Abnormal p65 nuclear translocation: Similarly, Sze et al. ‘s findings showed abnormal upregulation of transferritin and ferritin in aging rat ovarian tissue, accompanied by decreased ovarian mass and decreased serum estradiol content. Mechanically consistent with the results of this study, the *NF-κB* pathway is activated and enhances the expression of p65 and activates downstream TNF-α and iNOS while downregulating GPX4 [30]. In addition, the results of Wang et al. suggest that defective expression of the BNC1 gene can trigger the ferroptosis pathway of NF2-YAP and, ultimately, induce primary ovarian insufficiency [31]. Not only that, ferroptosis has also been reported to promote ferroptosis in polycystic ovary disease through iron-mediated mitochondrial autophagy activation of the TFRC-PINK1 signaling pathway [32]. It is not difficult to see that ferroptosis plays an important role in ovarian development, disease, and aging. In this study, we explored the protective mechanism of melatonin against ferroptosis of GCs and found that melatonin can inhibit the activation of the *NF-κB* pathway, inhibit the nucleation of p65, and inhibit ovarian granulosa cell death.

The important question emerging from our interventional experiments pertains to the precise functional relationship between NF-κB and PTGS2 within this ferroptotic cascade. While our data show that inhibiting NF-κB nuclear translocation (with DHMEQ) reduces PTGS2 expression, and that rescuing ferroptosis by targeting PTGS2 (with NS-398 or siPTGS2) does not obviously affect NF-κB activation, this correlative evidence primarily suggests a potential unidirectional regulatory relationship where NF-κB may act upstream of PTGS2. This putative axis is plausible given the well-established role of NF-κB as a transcriptional activator of PTGS2 in other pathological contexts, such as inflammation [33] and cancer [34]. However, the exact mechanistic link in porcine granulosa cells under ferroptosis stress remains to be fully elucidated. Future studies are necessary to conclusively determine whether the NF-κB p65 subunit directly binds to the PTGS2 gene promoter region in this specific model. Therefore, while our findings are consistent with a model where NF-κB regulates PTGS2 expression, contributing to ferroptosis execution, this proposed pathway warrants further in-depth validation. Furthermore, our data strongly support that melatonin’s protective effect is mediated through its ROS-scavenging capacity. As shown in our results, erastin-induced ferroptosis led to a dramatic accumulation of ROS and lipid peroxides (MDA), which was concomitant with mitochondrial dysfunction (reduced ΔΨm) and cell death. Melatonin treatment potently reversed these changes, normalizing ROS/MDA levels and preserving mitochondrial integrity (Figure 7). This suggests that the inhibition of the NF-κB/PTGS2 axis by melatonin is likely a consequence of reduced oxidative stress, as NF-κB is known to be activated by high ROS environments [35,36]. Therefore, we propose a mechanistic cascade whereby melatonin first neutralizes the excessive ROS generated during the ferroptotic process. This reduction in oxidative stress then mitigates the activation of the NF-κB pathway, leading to decreased PTGS2 expression, ultimately breaking the cycle of lipid peroxidation and preventing ferroptotic cell death. This places melatonin’s antioxidant activity upstream of the signaling pathway we identified, providing a more integrated understanding of its protective role. However, the precise temporal and causal relationships within this proposed cascade warrant further investigation. Future studies employing time-course experiments and specific inhibitors targeting ROS, NF-κB, and PTGS2 individually will be crucial to definitively establish this hierarchy of events.

This research is subject to certain limitations. The conclusions are derived solely from melatonin intervention; whether other antioxidants similarly inhibit granulosa cell ferroptosis via the NF-κB/PTGS2 axis remains unverified. Furthermore, the high cost of melatonin administration in large animal models may restrict its practical application. Future studies should validate the mechanism using diverse antioxidants and develop more economically viable strategies for clinical and agricultural translation.

## 5. Conclusions

**Figure 9:**
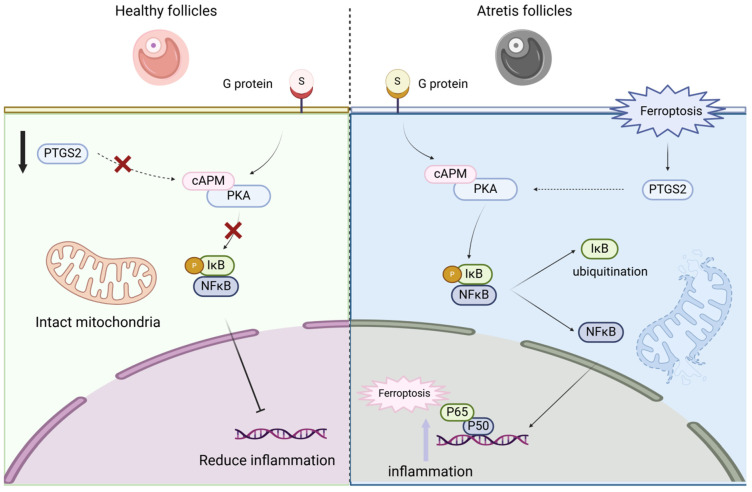
Ferroptosis mediates the *NF-κB/PTGS2* axis to regulate ovarian granulosa cell death.

This study explored the mechanism of cell apoptosis caused by ferroptosis between healthy follicles and atretic follicles. We activated cell ferroptosis by using erastin, a ferroptosis agonist, and then used the antioxidant melatonin to antagonize the cell function damage caused by the ferroptosis agonist and verify the NF-κB/PTGS2 regulatory pathway. We found that there were differences in the degree of ferroptosis between healthy follicles and atretic follicles, and the differential genes were mainly enriched in pathways such as cell cycle, amino acid metabolism, inflammatory response, and cysteine metabolism; by using erastin to induce ovarian granulosa cell death, we found that erastin-induced cell death played a role by activating the NF-κB/PTGS2 regulatory axis, and the use of NF-κB nuclear entry inhibitors could rescue the ferroptosis effect of erastin on cells; adding melatonin could inhibit the NF-κB/PTGS2 regulatory axis, thereby inhibiting the expression of downstream inflammatory factors and ferroptosis marker genes, and protecting ovarian granulosa cells (Figure 9). This work establishes ferroptosis as a pivotal mechanism in granulosa cell death and identifies melatonin as a potent therapeutic agent that rescues GCs by disrupting the NF-κB/PTGS2 pathway. These insights offer novel strategies to combat follicular atresia, with significant implications for improving ovarian function and reproductive outcomes in agriculture and medicine.

## Figures and Tables

**Table 1:** Sequence details of PTGS2 interference.

The Interfering Chain of PTGS2	Sequence
si-ssc-PTGS2_001	CCACGAGTACAACTATCAA
si-ssc-PTGS2_002	GCACTTCACCCATCAGTTT
si-ssc-PTGS2_003	CACCAACTTACAATATGCA

**Table 2:** Criteria for evaluating the degree of follicular atresia.

Follicular State	Diameter	P4/E2	Color	Vascular Distribution	Transparency
Healthy follicle	4–5 mm	≤5	pink	more	transparent
Primary atretic follicle	4–5 mm	5–10	light pink	less	translucency
Atretic follicles	4–5 mm	≥10	milky white and yellow	none	turbid

**Table 3:** Primer sequence.

Gene Accession Number	Primer	Sequence (5′→3′)
NM_214407	GPX4-F	GAGGCAAGACCGAAGTAAACTAC
	GPX4-R	CCGAACTGGTTACACGGGAA
NM_214001	TFRC-F	ACCATTGTCATATACCCGGTTCA
	TFRC-R	CAATAGCCCAAGTAGCCAATCAT
XM_005660803	FTH1-F	ACTTTGACCGCGATGATGTG
	FTH1-R	GCTCTCCCAGTCATCACAGT
NM_001244131	FTL-F	ATGGGGTGCGGACTTAGAAAG
	FTL-R	CTTGCGGTCTCTTCAGGGTAG
XM_005673820	ACSL4-F	CATCCCTGGAGCAGATACTCT
	ACSL4-R	TCACTTAGGATTTCCCTGGTCC
Z35483	IκBα-F	TGACCTTGTTCACGGGTCTG
	IκBα-R	TCTTGGACTCCGTTCCCTCT
NM_001114281	p65-F	GGCACCGGATTGAGGAGAAA
	p65-R	GCCTCTGTCAGTGTCCCTTC
GQ339058	TNFα-F	CAAGCCACTCCAGGACCC
	TNFα-R	GAAAACGTTGGTGGAAGGGC
NM_213821	SLC7A11-F	GACATCCGAGAAGCCAACAT
	SLC7A11-R	AGGTTGTTCCTGGGGAAGAT
NM_214131	caspase 3-F	GCCATGGTGAAGAAGGAAAA
	caspase 3-R	CACGCCATGTCATCTTCAGT
AM233489	BAX-F	AAGCGCATTGGAGATGAACT
	BAX-R	AAAGTAGAAAAGCGCGACCA
XM_021099602	Bcl2-F	GACTTTGCCGAGATGTCCAG
	Bcl2-R	ACGCTCTCCACACACATGAC
NM_213824	p53-F	CCTCACCATCATCACACTGG
	p53-R	GGCTTCTTCTTTTGCACTGG
XM_013977858	p21-F	GAGAGCGATGGAACTTCGAC
	p21-R	GGACAGCAACAGGTCCACAT
XM_021086047	ACTB-F	GGCTGTATTCCCCTCCATCG
	ACTB-R	CCAGTTGGTAACAATGCCATGT

## Data Availability

All other data will be made available upon reasonable request.

## Abbreviations

The following abbreviations are used in this manuscript:

LH	luteinizing hormone
FSH	follicle-stimulating estrogen
PCD	programmed cell death
EA	primary atretic follicles
PA	atretic follicles
H	healthy follicles
GC	granular cells
ROS	reactive oxygen species
GSH	glutathione
ALOX	arachidonate lipoxygenase
MUFA	monounsaturated Fatty Acids
PUfas	polyunsaturated fatty acids
